# Early mobilization of mechanically ventilated patients in the intensive care unit

**DOI:** 10.1186/s40560-016-0179-7

**Published:** 2016-07-29

**Authors:** Shunsuke Taito, Nobuaki Shime, Kohei Ota, Hideto Yasuda

**Affiliations:** 1 Division of Rehabilitation, Department of Clinical Practice and Support, Hiroshima University Hospital, 1-2-3, Kasumi, Minami-ku, Hiroshima 734-8551 Japan; 2 Department of Emergency and Critical Care Medicine, Institute of Biomedical and Health Sciences, Hiroshima University, 1-2-3, Kasumi, Minami-ku, Hiroshima 734-8553 Japan; 3 Department of Intensive Care Medicine, Kameda Medical Center, 929, Higashi-cho, Kamogawa, Chiba 296-8602 Japan

**Keywords:** Early mobilization, Mechanically ventilated patients, Intensive care unit, Adverse event, Rehabilitation

## Abstract

Several recent studies have suggested that the early mobilization of mechanically ventilated patients in the intensive care unit is safe and effective. However, in these studies, few patients reached high levels of active mobilization, and the standard of care among the studies has been inconsistent. The incidence of adverse events during early mobilization is low. Its importance should be considered in the context of the ABCDE bundle. Protocols of early mobilization with strict inclusion and exclusion criteria are needed to further investigate its contributions.

## Background

Early mobilization includes activities such as sitting, standing and ambulation, as well as passive exercises, like range of motion exercises and ergometry [1–3]. The term “early” has yet to be defined, since among the various studies, the onset of interventions may vary by as much as 1 week [1–7]. Mobilization in the intensive care unit (ICU) is generally considered early.

After the report by Schweickert et al., in 2009, of the effectiveness of early rehabilitation interventions on the physical and mental functions of mechanically ventilated patients [2], several studies have reported similar results in patients hospitalized in the ICU. However, studies of active mobilization beyond the sitting position are few [8, 9], and a consensus has been reached with respect to neither the timing of “early mobilization” [10, 11] nor the prescription of standardized interventions.

This review examines the protocols, the inclusion and exclusion criteria, the effectiveness and safety, and the obstacles to the implementation of early mobilization of mechanically ventilated patients in the ICU.

### Functional prognosis of mechanically ventilated patients

In a recent worldwide epidemiological survey, the survival rate of patients hospitalized in the ICU who met the diagnostic criteria of acute respiratory distress syndrome (ARDS) was increased up to 66 % by mechanical ventilation [12]. Another study found that nearly 70 % of patients presenting with acute respiratory failure who used mechanical ventilators were discharged from the ICU alive [13]. This increase in survival rate raised the issues of functional prognosis and quality of life (QOL) of the survivors. At 5 years after their discharge from the ICU, the exercise capacity of patients with ARDS remained lower than that of healthy controls, and approximately one fourth had difficulty returning to work [14].

The long-term use of mechanical ventilators may be a risk factor and a cause of ICU-acquired weakness [15], which has been observed in one fourth of patients requiring >7 days of mechanical ventilation [16]. Excessive immobilization is a major cause of ICU-acquired weakness [17], and a relationship between muscle weakness and duration of immobilization has been observed in patients with acute lung injury, whose muscle strength at the time of hospital discharge and 2 years later was reduced by 3 and 11 %, respectively, per each day of immobilization [18]. Therefore, patients mechanically ventilated in the ICU are likely to benefit from early mobilization to prevent ICU-acquired weakness, maintain long-term function, and preserve QOL.

### Effectiveness of early mobilization

While early mobilization has become easier to implement, few randomized trials have examined its effectiveness in mechanically ventilated patients (Table 1). In a landmark study, Schweickert et al. randomly assigned 104 mechanically ventilated patients to early physical and occupational therapy versus usual care, and compared the proportions of patients in each group who returned to independent functional status at the time of discharge from the hospital [2]. An independent functional status at hospital discharge was regained by 59 % of patients in the intervention group, in whom early mobilization began at a mean of 1.5 days after the onset of mechanical ventilation, compared with 35 % of patients in the control group in whom early mobilization began at a mean of 7.4 days (*P* = 0.02). Patients in the early mobilization group also suffered from shorter periods of delirium and required fewer days of recurrent mechanical ventilation than the control group during 28 days of follow-up. Burtin et al. evenly assigned 90 mechanically ventilated patients to (a) a 20-min session of bicycle ergometer exercise daily, 5 days/week, in addition to standard care, versus (b) standard care only, and compared the outcomes of 6-min walk tests at the time of discharge from the hospital [3]. In the intervention group, the median 196 m covered in 6 min was significantly longer than the median 143 m covered in the control group. Furthermore, physical function ascertained by the 36-item Short-Form Health Survey were significantly greater in the intervention than in the control group, and the quadriceps femoris strength at discharge were significantly increased in the intervention group, but not in the control group.

**Table 1:** Randomized studies of the effects of early mobilization

Reference #	*n*	Study group		Days between intubation and onset of		Outcomes
		Intervention	Control	Intervention	Control	
[2]	104	Exercise and mobilization	Standard care	1.5	7.4	Primary: number of patients returning to independent functional status (ability to perform 6 daily activities and walking independently) at time of discharge from hospital. Secondary: (1) number of hospital days with delirium (2) number of ventilator-free days during first 28 days of hospitalization (3) length of stay in ICU and in hospital
[3]	90	Usual care + bicycle ergometer, 20 min/day, at an intensity level adjusted individually ×5 days/week	Respiratory therapy adjusted to the individual needs + standardized sessions of upper and lower extremities mobilization 5 days/week	14	10	Primary: distance covered in 6 min at time of discharge from the hospital Secondary: isometric quadriceps strength and functional status
[4]	150	Mechanically ventilated patient: physical therapy 15 min/day Non-mechanically ventilated patients: physical therapy 2 × 15 min Exercises: walking in place, moving from sitting to standing, arm and leg active and active resistance motion	Physical therapists provided respiratory and mobility management, based upon individual patient assessment according to unit protocols Usual care was available 7 days/week, 12 h/day	5	5	Primary: distance covered in 6 min at 12 months Secondary: Timed Up and Go Test, physical function in ICU test, assessment of QOL Instrument utility and short form 36 health survey
[5]	120	Delivered for 30 min/day, 7 days/week, while in ICU. Intensive physical therapy program included: 1. Proper breathing techniques during exercise 2. Progressive range of motion; 3. Muscle strengthening exercises 4. Exercises to increase core mobility and strength 5. Retraining of functional mobility	Standard of care physical therapy programs based on national survey Range of motion exercises, positioning, and functional mobility retraining 3 days/week for 20 min in ICU	8	8	Primary: short form of the continuous scale physical functional performance test at 4 weeks Secondary: number of ICU- and hospital-free days on day 28; discharged home, all-cause mortality on day 28, and institution-free days on days 90 and 180
[6]	50	Early goal-directed mobilization comprised functional rehabilitation treatment at the highest level of activity possible for that patient assessed by the ICU mobility scale while receiving mechanical ventilation.	Not based on protocol All usual unit practices were continued, without restrictions to physical therapy or sedation practice	3	3	Primary: higher maximum level of activity measured using the ICU mobility scale, increased duration of activity measured in min/day during the ICU stay compared with standard care Secondary: time from admission to first mobilization; duration of mechanical ventilation, ICU and hospital length of stay, and overall duration of hospitalization; serious adverse events, number of ventilator- and ICU-free days on day 28; measurement of physical function with the physical function in ICU, the functional status score in ICU, and the Medical Research Council Manual Muscle Tests; ICU-acquired weakness
[7]	300	Standardized rehabilitation therapy 7 days a week from enrollment through hospital discharge protocol contained 3 exercise types: passive range of motion, physical therapy, and progressive resistance exercises	Usual care; received routine care as dictated by the patient’s attending physician from Monday through Friday	1	7	Primary: hospital length of stay Secondary: Short Performance Physical Battery score, muscular strength, short form Functional Performance Inventory score, 36-Item Short Form Health Survey physical health survey and mental health survey, mini-mental state examination score (measure of physical function were obtained at ICU discharge, hospital discharge and 2, 4, and 6 months after enrollment, health-related quality-of-life measures were obtained at hospital discharge and 2,4, and 6 months after enrollment)

Other studies, however, have not confirmed the efficacy of early mobilization. Two randomized trials including >100 mechanically ventilated, critically ill patients, observed insignificant improvements in physical function after intensive physical therapy [4, 5]. More recent, single-center randomized controlled trial, including 300 patients cared in ICU with acute respiratory failure requiring mechanical ventilation, has reported that daily standardized rehabilitation therapy consisting of passive range of motion, physical therapy, and progressive resistance exercise did not result in decreased duration of mechanical ventilation, hospital or ICU length of stay, and long-term physical function in comparison with usual care [7]. Another small, randomized pilot trial reported a significant increase in activity level in an intervention group after undergoing gait training in the ICU, though the length of stay in the ICU and the activity level 6 months later were similar in both study groups [6].

Differences in the interventions imposed in both groups may explain the insignificant effect of early mobilization. In one “negative” study, only 52 % of the planned participants in the intervention group were mobilized early, and 52 % of the patients assigned to the usual care group were mobilized early out of bed [19]. In addition, the time to first intervention or the interventions performed before randomization may influence the study results. In another negative study, the interventions began after eight ventilator days and detailed information regarding the intensity of physical therapy before randomization was not specified [5, 20].

Further studies and analyses are needed to accurately measure the effectiveness of early mobilization, where the contents of “standard care” and the length of the intervention are clearly defined.

### Early mobilization in the ABCDE bundle

The “ABCDE bundle” is a strategy incorporated awakening and breathing coordination, delirium monitoring/management, and early exercise/mobility. It was proposed by Vasilevskis et al. in 2010, aiming at improving the prognosis of mechanically ventilated patients in the ICU by preventing delirium and ICU-acquired weakness [21]. The application of all steps, from A to E, to critically ill patients facilitates early mobilization as a voluntary activity during optimal sedation and analgesia. The implementation of the ABCDE bundle shortens the time spent on the ventilator, decreases the incidence of delirium, and increases the rate of early ambulatory mobilization practice [22]. A survey submitted in the state of Michigan in the USA revealed that early mobilization was adopted by 64 % of hospitals, though only 12 % included the whole ABCDE bundle [23]. Standing, walking, and gait exercises can reach higher levels of performance when whole ABCDE bundles are practiced. It is noteworthy that performing the A to D bundle is a prerequisite in order to effectively achieve early mobilization.

### Adverse events

The incidence of adverse events during early mobilization is shown in Table 2. Although the majority of studies reported a <5 % incidence of adverse events [2–6, 24–28], it reached 16 % in one study [29], perhaps because of differences in the definitions of adverse events. Some studies have reported fatal adverse events, including extubation or desaturation; however, early mobilization is generally safe.

**Table 2:** Adverse events during early mobilization

Reference #	*n*	Early mobilization intervention	Incidence of adverse events	Adverse events
[24]	103	Active mobilization: 1449 sessions: sitting on bed and in chair, ambulation	0.96 %	Fall, systolic blood pressure <90 mmHg, oxygen desaturation, feeding tube extraction, systolic blood pressure >200 mmHg
[3]	90	Passive and active therapy, bicycle ergometer exercise	3.76 %	SpO_2_ < 90 %, systolic blood pressure >180 mmHg, >20 % decrease in diastolic blood pressure
[2]	104	Passive and active range of motion, sitting, balance exercises, activities of daily living, transfer training, walking	4.0 %	Patient instability (most often because of perceived patient-ventilator asynchrony), 0.2 % serious (desaturation <80 %)
[29]	99	Active mobilization: 498 sessions	16 %	Desaturation ≧5 %, heart rate increase >20 %, ventilator asynchrony/tachypnea, agitation/discomfort, device removal
[25]	20	Active mobilization: 424 sessions: chair sitting, head up tilt, walking	3 %	Decreased muscle tone, hypoxemia, extubation, orthostatic, hypotension
[4]	150	Walking in place, sit to stand transfers, arm and leg active range of motion	None major	–
[26]	1110	Active mobilization: 5267 session: in-bed exercise, in-bed bicycling, sitting, transfer, standing, walking	0.6 %	Arrhythmia, MAP > 140 mmHg, MAP < 55 mmHg, oxygen desaturation, fall, feeding tube extraction, radial artery catheter removal, chest tube removal
[27]	637	16-level early progressive mobility protocol	Not validated	–
[28]	99	Active mobilization: 520 sessions	5 %	Respiratory distress, desaturation, tachypnea or bradycardia, patient’s intolerance, tracheostomy removal
[5]	120	Proper breathing techniques during exercise, progressive range of motion, muscle strengthening exercises, exercises designed to improve core mobility and strength, functional mobility retraining	0.16 %	Syncopal episode during a PT session, readmitted to the hospital with polyarthralgia
[6]	50	Early goal-directed mobilization	0.96 %	Fall, systolic blood pressure <90 mmHg, oxygen desaturation, feeding tube extraction, systolic blood pressure >200 mmHg
[7]	300	Passive range of motion, physical therapy, and progressive resistance exercises	6.0 %	Deaths, device removals, reintubations, and patient falls during physical therapy

### Inclusion and exclusion criteria and protocols

Each study of early mobilization in the ICU chooses independently its inclusion/exclusion criteria. In addition, protocols of early mobilization are inexistent, including in hospitals where it is being practiced [30]. Consensus statements regarding the performance of exercise by mechanically ventilated patients [31], or risk categories and safety criteria have been proposed in clinical guidelines of physical therapy and rehabilitation for patients in ICU [32]. Since 2014, the Early Rehabilitation Committee of the Japanese Society of Intensive Care Medicine has developed an “evidence-based expert consensus” early rehabilitation manual. To date, only 16 % of healthcare providers have prepared protocols of early mobilization, and 36 % are planning to develop a protocol [10, 11]. A survey submitted in the USA found that the adoption of early mobilization protocols shortens the time needed to regain a higher level of ambulatory mobility [1, 33].

Furthermore, a 2013 survey conducted in 12 ICU in Australia and New Zealand found that among 1395 sessions of physical therapy in 192 patients, active mobilization during mechanical ventilation was used only 315 times in the absence of protocol [34]. Based on these observations, Hodgson et al. conducted a randomized trial with a preliminary protocol intervention program, called early goal-directed mobilization, in order to promote the active mobilization of mechanically ventilated patients. This program aimed at conducting the highest level of 30–60 min interventions based on the evaluation of ICU mobility scale [6]. Compared to the usual care, the intervention group reached higher levels of active mobilization and longer duration of active mobilization. Secondary endpoints, such as health-related QOL, anxiety, depression, activity of daily living levels, and rates of return to work were similar in both groups. A study including >500 participants is needed to evaluate patient-centered measures as primary outcome. Hospitals which had already implemented early mobilization found no significant differences in frequency of early mobilization regardless of early mobilization protocols [30], suggesting that, in hospitals that are already practicing early mobilization, protocols are of uncertain efficacy.

### Current status and further studies

Although early mobilization is a safe and effective procedure (Table 2), surveys performed at multiple sites have revealed that active mobilization beyond sitting is not commonly practiced, and that it varies among countries.

A survey conducted in 38 Australian and New Zealander ICU in 2009 and 2010 revealed that exercise was limited to the bed in 28 % of 514 patients, and that 25 and 18 %, respectively, performed standing and walking exercises, while no standing and walking exercises were performed by mechanically ventilated patients [9]. Another survey conducted in 2010 and 2011, reported that only 60 % of patients in Australian and 40 % in Scottish ICU reached a level of active mobilization higher than sitting [35], while in 116 German ICU in 2011, 185 of 783 mechanically ventilated (24 %) and only 8 % of tracheally intubated patients reached a level of early mobilization higher than sitting [8]. In the American state of Washington, a questionnaire submitted in 2012 and 2013 revealed that a wide range of motion exercises was routinely practiced in >70 % of hospitals, while only approximately 10 % conducted sitting and standing exercises routinely [33]. In contrast, a survey submitted to Japanese providers of intensive care revealed that range of motion exercises are often practiced, including sitting and standing exercises in 60 and 40 % of patients, respectively [10, 11]. Further studies are warranted to evaluate the effects of early mobilization in Japanese ICU, where extensive exercises are widely practiced.

### Impediments and strategies

Based on 40 previous studies, Dubb et al. identified 28 obstacles in the way of early mobilization, including 14 (50 %) related to patients; five structural barriers (18 %), five related to the cultures of ICU (18 %); and four process-related impediments (14 %) [36]. They offered >70 solutions or strategies to deal with each barrier. The obstacles to early mobilization may vary depending on the physician(s), nurse(s), and physical therapist(s) involved in the care of each patient [37, 38]. Inter-professional collaboration needs to be developed with a view to create educational programs and research projects to address the challenges represented by the early mobilization of mechanically ventilated patients in the ICU.

## Conclusions

Despite multiple recent studies claiming the safety and effectiveness of early mobilization of mechanically ventilated patients, convincing trials remain few. Early has not been accurately defined, and the differences between intervention and standard care vary among studies. The methods and frequency of standardized early mobilization of mechanically ventilated patients remain unsettled. In addition, the number of the studies included is not big enough and their sample sizes are limited. The generalizability of the findings in this review would therefore be open to question. Additional clinical trials are needed to confirm the efficacy of early mobilization of mechanically ventilated patients in the ICU.

## Abbreviations

ARDS, acute respiratory distress syndrome; ICU, intensive care unit; QOL, quality of life
